# Direct Regulons of AtxA, the Master Virulence Regulator of Bacillus anthracis

**DOI:** 10.1128/mSystems.00291-21

**Published:** 2021-07-20

**Authors:** Yoshikazu Furuta, Cheng Cheng, Tuvshinzaya Zorigt, Atmika Paudel, Shun Izumi, Mai Tsujinouchi, Tomoko Shimizu, Wim G. Meijer, Hideaki Higashi

**Affiliations:** a Division of Infection and Immunity, Research Center for Zoonosis Control, Hokkaido University, Sapporo, Hokkaido, Japan; b UCD School of Biomolecular and Biomedical Science, University College Dublin, Dublin, Ireland; c Department of Medical Laboratory Science, Faculty of Health Sciences, Hokkaido University, Sapporo, Hokkaido, Japan; University of California, San Francisco

**Keywords:** *Bacillus anthracis*, Cappable-seq, ChIP-seq, DNA-binding proteins, small RNA, transcription factors, transcriptional regulation, virulence factors

## Abstract

AtxA, the master virulence regulator of Bacillus anthracis, regulates the expression of three toxins and genes for capsule formation that are required for the pathogenicity of B. anthracis. Recent transcriptome analyses showed that AtxA affects a large number of genes on the chromosome and plasmids, suggesting a role as a global regulator. However, information on genes directly regulated by AtxA is scarce. In this work, we conducted genome-wide analyses and cataloged the binding sites of AtxA *in vivo* and transcription start sites on the B. anthracis genome. By integrating these results, we detected eight genes as direct regulons of AtxA. These consisted of five protein-coding genes, including two of the three toxin genes, and three genes encoding the small RNAs XrrA and XrrB and a newly discovered 95-nucleotide small RNA, XrrC. Transcriptomes from single-knockout mutants of these small RNAs revealed changes in the transcription levels of genes related to the aerobic electron transport chain, heme biosynthesis, and amino acid metabolism, suggesting their function for the control of cell physiology. These results reveal the first layer of the gene regulatory network for the pathogenicity of B. anthracis and provide a data set for the further study of the genomics and genetics of B. anthracis.

**IMPORTANCE** Bacillus anthracis is the Gram-positive bacterial species that causes anthrax. Anthrax is still prevalent in countries mainly in Asia and Africa, where it causes economic damage and remains a public health issue. The mechanism of pathogenicity is mainly explained by the three toxin proteins expressed from the pXO1 plasmid and by proteins involved in capsule formation expressed from the pXO2 plasmid. AtxA is a protein expressed from the pXO1 plasmid that is known to upregulate genes involved in toxin production and capsule formation and is thus considered the master virulence regulator of B. anthracis. Therefore, understanding the detailed mechanism of gene regulation is important for the control of anthrax. The significance of this work lies in the identification of genes that are directly regulated by AtxA via genome-wide analyses. The results reveal the first layer of the gene regulatory network for the pathogenicity of B. anthracis and provide useful resources for a further understanding of B. anthracis.

## INTRODUCTION

AtxA is the master virulence regulator of Bacillus anthracis, the Gram-positive bacterial species that causes anthrax in humans and animals. Following the growth of B. anthracis under high-CO_2_/bicarbonate conditions at 37°C, AtxA regulates the transcription of the B. anthracis virulence genes. The three anthrax toxin genes, *pagA* (protective antigen), *cya* (edema factor), and *lef* (lethal factor), are present on the pathogenicity island of the pXO1 plasmid and specifically activated by AtxA and high-CO_2_/bicarbonate conditions (1–8). *pagA* is cotranscribed with *pagR*, and PagR regulates not only *pagAR* but also chromosomal genes of S-layer proteins, *sap* and *eag* (9, 10). AtxA indirectly regulates the capsule biosynthetic operon *capBCADE* on the pXO2 plasmid via the upregulation of other regulatory genes on pXO2, *acpA* and *acpB* (8, 11–15). Systematic analysis of transcriptomes using microarrays and transcriptome sequencing (RNA-seq) revealed that AtxA regulates not only the toxin and capsule genes but also many other genes on the chromosome and the plasmids, suggesting a role as a global regulator (16–19).

Biochemical studies and the crystal structure of AtxA suggested that AtxA multimerizes and binds to DNA at the two helix-turn-helix motifs at the N terminus (20–22). AtxA was also reported to form a heteromultimer with AcpA, one of its regulons on pXO2, and downregulate a gene for capsule formation (19). Another critical factor for AtxA-binding activity is the phosphorylation of two histidine residues (20, 22–24). The phosphorylation of H199 has been suggested to maintain the activity of AtxA for the induction of *pagA in vivo* and the binding of AtxA to the promoter region of *pagA in vitro* (20, 24). Another histidine residue, H379, was shown to inhibit AtxA activity by affecting its dimer formation (22).

However, the molecular mechanism of target recognition and gene regulation by AtxA is not understood in detail. As AtxA is highly likely to function as a transcription factor, several studies have been attempted to detect the specific target of AtxA for DNA binding. Sequences upstream of the transcription start sites (TSSs) of the toxin genes and some other regulons have been analyzed (2, 3, 25), but no specific DNA motifs were noted (26). The bending of promoter regions, rather than DNA sequence motifs, was also suggested to be a target of AtxA (27). Recent work revealed that AtxA directly binds to the promoter region of *pagA* and suggested the importance of a stem-loop structure of DNA for AtxA binding (24). Another report also showed indirect evidence of AtxA binding to the upstream region of *pagA* (28). Other than the promoter region of *pagA*, there was little information about AtxA-binding regions, and it was unknown which AtxA regulons were directly regulated by AtxA binding at their promoter. Such information is important to understand the mechanism of virulence induction of B. anthracis and the functions of AtxA in detail.

To understand which genes are directly regulated by AtxA, we conducted two genome-wide analyses: chromatin immunoprecipitation sequencing (ChIP-seq) for the detection of AtxA-binding sites *in vivo* and Cappable-seq for the systematic detection of transcription start sites. Eight genes were detected with both AtxA binding at their 5′ untranslated regions (UTRs) and upregulation of the transcription of their TSSs. Here, we considered these genes direct regulons of AtxA.

## RESULTS

### AtxA binds to 11 sites in the B. anthracis genome.

For the genome-wide detection of regions that were bound by AtxA *in vivo*, ChIP-seq was conducted using the B. anthracis 34F2 (pXO1^+^ pXO2^−^) wild-type (WT) strain and a derivative strain that expresses AtxA fused with a 1× FLAG tag at the C terminus from pXO1; FLAG-tagged AtxA was previously shown to have minimal effects on the activity of AtxA (19, 21). Both WT and AtxA-tagged strains were grown under high-CO_2_/bicarbonate conditions (15% CO_2_ and 0.8% NaHCO_3_), and log-phase cultures were collected and applied for ChIP-seq.

We detected 11 sites with significant enrichment of reads by selection with anti-FLAG antibody and considered these *in vivo* AtxA-binding sites (Table 1). Among them, 10 sites were detected on plasmid pXO1, and only 1 was detected on the chromosome. Binding sites on pXO1 showed enrichment, especially within the region of the pathogenicity island, suggesting that AtxA function is related to the regulation of genes in the pathogenicity island of pXO1.

**TABLE 1:** AtxA-binding regions^*a*^

Binding site	Replicon	ChIP-seq			Cappable-seq			Gene	Old locus tag
		Start position	End position	Width (bp)	Whole FDR	TSS	TSS log_2_ FC		
AtxA_BS_01	pXO1	105401	106100	700	5.1E−06	105924 (−)	4.16	*xrrB*	NA
AtxA_BS_02	pXO1	106351	106850	500	6.6E−03	106705 (+)	4.80	GBAA_RS28950 (S-layer protein)	GBAA_pXO1_0124
AtxA_BS_03	pXO1	110651	111300	650	2.3E−05	NA	NA	NA	NA
AtxA_BS_04	pXO1	119401	119800	400	3.9E−09	119551 (−)	4.38	GBAA_RS29020 (hypothetical protein)	GBAA_pXO1_0137
AtxA_BS_05	pXO1	129301	129600	300	3.6E−03	129517 (+)	4.77	GBAA_RS29065 (hypothetical protein)	GBAA_pXO1_0148
AtxA_BS_06	pXO1	131251	131450	200	5.0E−05	131385 (+)	4.55	*xrrA*	NA
AtxA_BS_07	pXO1	141601	141950	350	5.1E−06	141844 (+)	5.05	*xrrC*	NA
AtxA_BS_08	pXO1	143201	144000	800	1.2E−05	143721 (+)	4.18	GBAA_RS29110 (*pagA*)	GBAA_pXO1_0164
AtxA_BS_09	pXO1	147501	147850	350	5.1E−06	NA	NA	NA	NA
AtxA_BS_10	pXO1	151601	152450	850	5.1E−05	151815 (−)	3.76	GBAA_RS29135 (*lef*)	GBAA_pXO1_0172
AtxA_BS_11	Chromosome	4964501	4964800	300	4.6E−08	NA	NA	NA	NA

a Whole FDR, false discovery rate for the whole AtxA-binding region; TSS, transcription start site; FC, fold change; NA, not applicable; (+), forward strand; (−), reverse strand.

**FIG 1:**
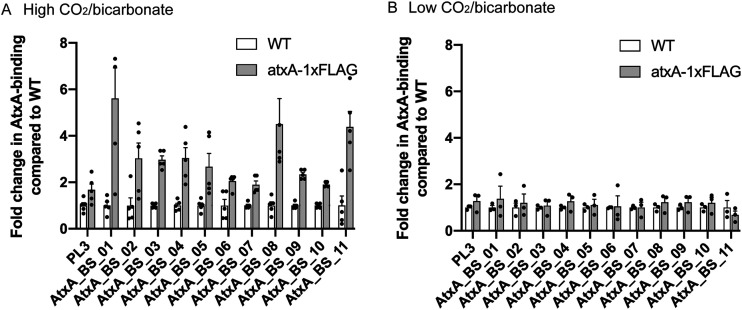
ChIP-qPCR. (A) ChIP samples isolated from cells grown under high CO_2_/bicarbonate levels (15% CO_2_ and 0.8% NaHCO_3_). (B) ChIP samples isolated from cells grown under low CO_2_/bicarbonate levels (no additional CO_2_ or bicarbonate). PL3 is the marker region of the B. anthracis chromosome that is not expected to bind with AtxA.

AtxA affects the expression of various genes, including the three toxin genes, when grown under high-CO_2_/bicarbonate conditions and is also expressed under low-CO_2_/bicarbonate conditions (29, 30). However, it was unknown whether AtxA, under low-CO_2_/bicarbonate conditions, binds to known target sites but fails to induce regulons or if it fails to bind to the target sites at all. To answer this and validate the ChIP-seq results, strains were grown under conditions of both high and low CO_2_/bicarbonate and assessed via ChIP-quantitative PCR (ChIP-qPCR) (Fig. 1). In the strains that grew under the high-CO_2_/bicarbonate conditions (15% CO_2_ and 0.8% NaHCO_3_), all 11 target sites showed enrichment after the selection of DNA fragments bound by AtxA, validating the ChIP-seq results (Fig. 1A). However, ChIP-qPCR for strains that grew under the low-CO_2_/bicarbonate conditions (without the addition of CO_2_ or NaHCO_3_) showed no enrichment (Fig. 1B). This difference suggested that AtxA does not induce the expression of target genes under the low-CO_2_/bicarbonate conditions because of a lack of binding to the target loci.

To analyze whether the AtxA-binding target could be characterized by specific sequence motifs and/or DNA bending, we used MEME (31) for sequence motif analysis of the AtxA-binding sites but did not discover any specific motifs. These sequences were assessed via bend.it (32) to calculate the intrinsic curvature of the regions; as a control, the curvatures of all genomic regions with a 500-bp sliding window and those of promoter regions were also calculated. AtxA-binding sites had a significantly higher distribution of bending parameters than both genomic regions and promoters (*P* value of <0.001 by a Wilcoxon test) (see Fig. S1 in the supplemental material). However, as many genomic regions and promoters without AtxA binding also showed a high level of intrinsic curvature, it was difficult to conclude that the curvature is the only factor for AtxA-binding sites.

10.1128/mSystems.00291-21.1 FIG S1 Comparison of DNA bending. (A) Comparison between the AtxA-binding region and the 500-bp sliding window of each amplicon. (B) Comparison between the AtxA-binding region and upstream regions of TSSs. Download FIG S1, PDF file, 0.9 MB. Copyright © 2021 Furuta et al. 2021 Furuta et al. https://creativecommons.org/licenses/by/4.0/ This content is distributed under the terms of the Creative Commons Attribution 4.0 International license.

### Genome-wide detection of TSSs in B. anthracis.

If AtxA functions as a transcription factor, the binding of AtxA would be followed by changes in transcription from TSS around the binding region. To elucidate such transcriptional regulation, we conducted Cappable-seq (33) to detect TSSs in a genome-wide manner. Cappable-seq can detect the position of TSSs at single-nucleotide resolution by the enrichment of the transcripts with triphosphate at the 5′ end, which is characteristic of bacterial RNA transcribed from TSSs. We applied this method to both B. anthracis 34F2 (pXO1^+^ pXO2^−^) WT and Δ*atxA* strains grown under high-CO_2_/bicarbonate conditions (15% CO_2_ and 0.8% NaHCO_3_) to catalog the TSSs in B. anthracis and detect the TSS regulated by the expression of AtxA.

**FIG 2:**
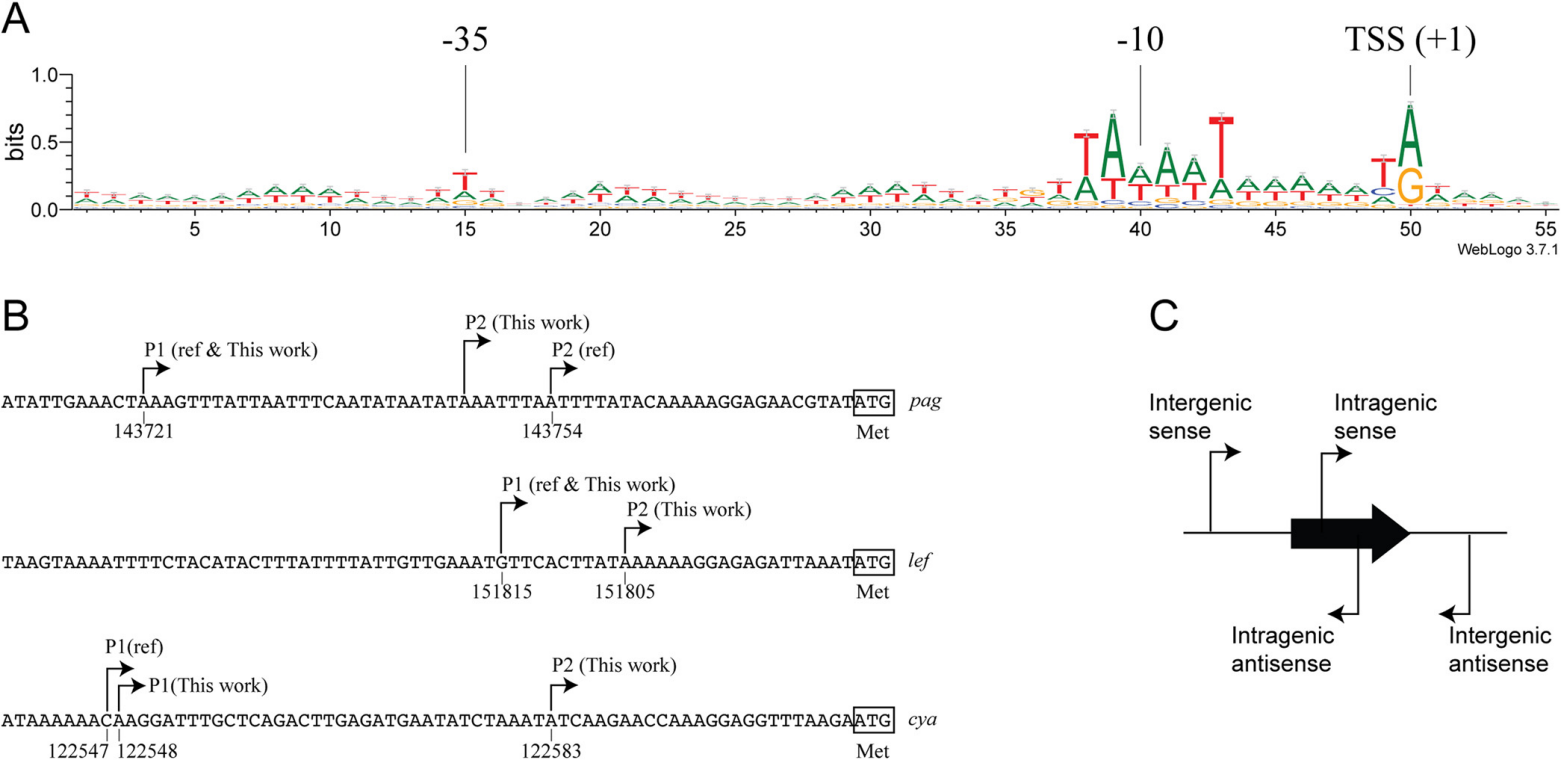
Sequence motifs and TSSs detected by Cappable-seq. (A) Results of WebLogo analysis of the region spanning positions −49 to +6 of all TSSs detected in the WT strain. (B) TSSs of the three toxin genes. The start codon of each gene is squared. “Ref” indicates TSSs detected in previous literature. See Table S1 in the supplemental material for the reference information. (C) Classification of TSSs in this work.

In total, we detected 6,088 and 5,857 TSSs for WT and Δ*atxA* strains, respectively (Data Set S1). To validate the identification of these TSSs, sequences around TSSs were assessed by sequence motif analysis to detect purine enrichment at the TSSs and AT-rich motifs at the −10 and −35 promoter boxes, which are typical characteristics of bacterial TSSs (Fig. 2A). We further compared our results with TSS positions that had been previously detected via primer extension or other methods (2, 3, 25, 34–39) (Table S1). The TSSs for 10 genes were also detected in at least one of the strains used here. Among these genes, five (*pagA*, *lef*, *atxA*, *xrrA*, and *abrB*) showed identical positions for their primary TSSs. Differences were present in the other five genes (*cya*, *xrrB*, *sap*, *eag*, and *alo*) but were within 10 nucleotides. For nonprimary TSSs, although our work failed to detect the P3 promoter of *atxA* (34), we found additional TSSs for *lef* and *cya*; we renamed the previously reported TSS P1 and named the secondary TSS P2 (Fig. 2B). Therefore, Cappable-seq and other methods were consistent in the detection of most of the TSSs and complemented each other in the detection of nonprimary TSSs.

10.1128/mSystems.00291-21.4 TABLE S1 Validation of TSSs with previous reports. Download Table S1, PDF file, 0.07 MB. Copyright © 2021 Furuta et al. 2021 Furuta et al. https://creativecommons.org/licenses/by/4.0/ This content is distributed under the terms of the Creative Commons Attribution 4.0 International license.

10.1128/mSystems.00291-21.6 DATA SET S1 TSSs detected in each strain. Download Data Set S1, XLSX file, 1.2 MB. Copyright © 2021 Furuta et al. 2021 Furuta et al. https://creativecommons.org/licenses/by/4.0/ This content is distributed under the terms of the Creative Commons Attribution 4.0 International license.

For the WT strain, 5,683 (93%) TSSs were detected on the chromosome, and 405 (7%) were detected on the pXO1 plasmid, while 5,504 (94%) and 353 (6%) were detected on the chromosome and pXO1 for the Δ*atxA* strain, respectively (Table 2; Tables S1 and S2). Considering the sizes of the chromosome (5.22 Mb) and pXO1 (181 kb), the number of TSSs showed significant enrichment on pXO1 (*P* < 10^−15^ for both strains by a chi-square test). We also classified each TSS by position and relative direction against the neighboring genes or the gene that the TSS was positioned within (Fig. 2C and Table 2). A total of 51% (WT) and 54% (Δ*atxA*) of the TSSs were positioned in an intergenic region and on the same strand as the downstream gene (intergenic sense), suggesting a function in mRNA production, whereas the remaining TSSs (intergenic antisense, intragenic sense, and intragenic antisense) may play a role in the transcription of unannotated genes, shorter secondary transcripts, or antisense RNA for gene regulation.

**TABLE 2:** Classification of TSSs

Type of TSS^*a*^	No. of TSS in strain					
	WT			Δ*atxA*		
	Chromosome	pXO1	Total	Chromosome	pXO1	Total
Intergenic sense	2,982	150	3,132	3,018	131	3,149
Intergenic antisense	458	75	533	421	73	494
Intragenic sense	1,499	112	1,611	1,420	97	1,517
Intragenic antisense	744	68	812	645	52	697

Total	5,683	405	6,088	5,504	353	5,857

a See Fig. 2C.

10.1128/mSystems.00291-21.5 TABLE S2 Primers. Download Table S2, PDF file, 0.06 MB. Copyright © 2021 Furuta et al. 2021 Furuta et al. https://creativecommons.org/licenses/by/4.0/ This content is distributed under the terms of the Creative Commons Attribution 4.0 International license.

**FIG 3:**
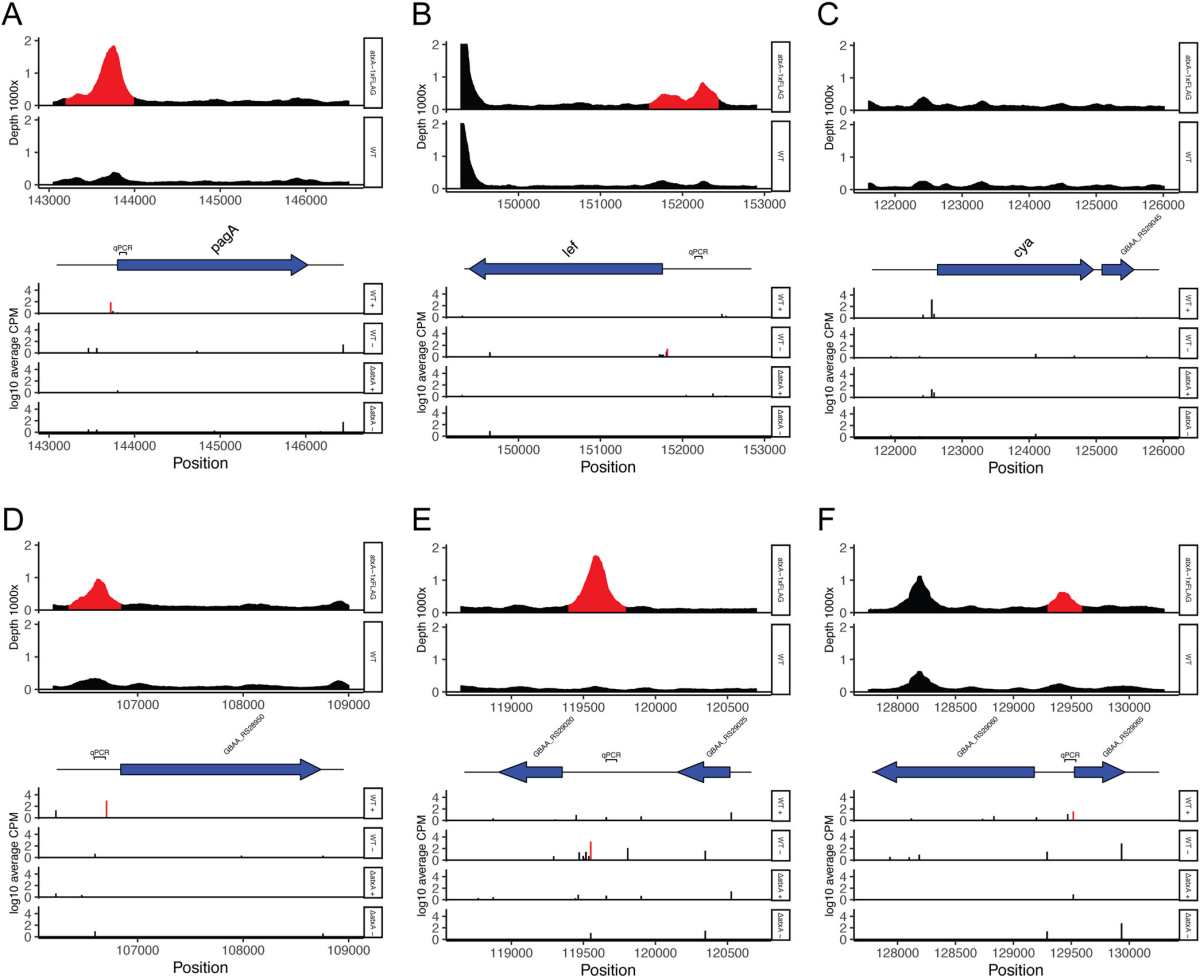
AtxA binding and TSS changes in AtxA direct regulons and toxin genes. (A) *pagA*; (B) *lef*; (C) *cya*; (D) GBAA_RS28950; (E) GBAA_RS29020; (F) GBAA_RS29065. The top panels show the ChIP-seq results; red indicates detected AtxA binding. The middle panels show the genomic context around the gene of interest. The bottom panels show the coverage of each position in Cappable-seq on the forward strand of the WT, the reverse strand of the WT, the forward strand of the Δ*atxA* strain, and the reverse strand of the Δ*atxA* strain, from the top. TSSs of direct regulons are shown in red. Protein-coding genes are represented by blue arrows.

Comparison of the WT and Δ*atxA* strains revealed a significant difference in the transcription levels of 311 TSSs. Among these, 177 TSSs were observed to have a significant increase in transcription in the WT strain, while 134 had a decrease in transcription (Data Set S1). These included the upregulation of TSSs of known AtxA regulons, such as the three toxin genes in the WT, which validated the results (Fig. 3A to C).

### AtxA directly regulates the transcription of five genes and three small RNAs.

We then compared the results for both AtxA-binding sites and TSSs from ChIP-seq and Cappable-seq, respectively, to deduce the genes directly regulated by AtxA. We considered a gene a direct regulon of AtxA when a TSS from which the gene was supposed to be transcribed fulfilled all the following criteria: (i) the TSS is included in the AtxA-binding region, (ii) the TSS was significantly upregulated in the WT strain, and (iii) the transcription level from the TSS was higher than the threshold (>16 average counts per million reads) and the highest among the TSSs around or within the AtxA-binding site. Among 11 AtxA-binding sites, 8 of them were found with a TSS that fulfilled these criteria, whereas the other 3 without such a TSS were not analyzed further.

Five AtxA-binding sites were detected with a TSS fulfilling these criteria with a downstream protein-coding gene. These were *pagA*, *lef*, GBAA_RS28950, GBAA_RS29020, and GBAA_RS29065. Two of the three toxin genes of B. anthracis were shown to be direct regulons of AtxA, as expected (Fig. 3A and B). However, one of the toxin genes, *cya*, was not observed with a significant signal of AtxA binding at the promoter region, although the upstream TSS was significantly upregulated by the presence of AtxA (Fig. 3C). We conducted ChIP-qPCR for the region around the TSS of *cya* and confirmed no enrichment by AtxA binding (Fig. S2). The other three genes were annotated to encode an S-layer protein (GBAA_RS28950) and two hypothetical proteins (GBAA_RS29020 and GBAA_RS29065); these were also shown to be upregulated by AtxA and/or under high-CO_2_/bicarbonate conditions (15% CO_2_ and 0.8% NaHCO_3_) in a previous transcriptome paper (16) (Fig. 3D to F). Although a range of genes were previously reported to be affected by AtxA expression (16–19), the number of regulons directly upregulated by AtxA seemed to be much smaller.

10.1128/mSystems.00291-21.2 FIG S2 ChIP-qPCR of the upstream region of *cya* for ChIP samples isolated from cells grown under high CO_2_/bicarbonate levels (15% CO_2_ and 0.8% NaHCO_3_). Download FIG S2, PDF file, 0.9 MB. Copyright © 2021 Furuta et al. 2021 Furuta et al. https://creativecommons.org/licenses/by/4.0/ This content is distributed under the terms of the Creative Commons Attribution 4.0 International license.

**FIG 4:**
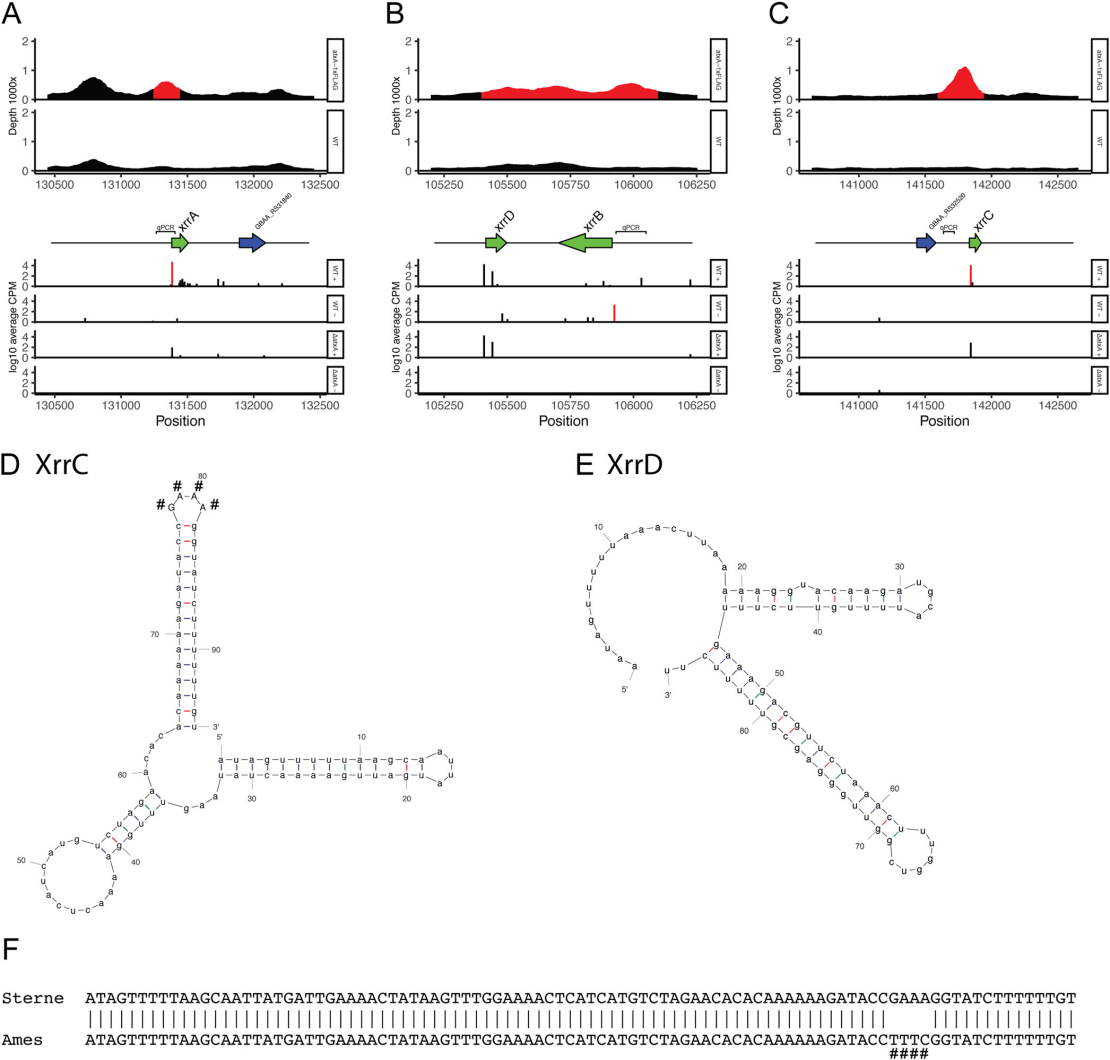
Small RNAs directly regulated by AtxA. (A to C) Integrated view of ChIP-seq and Cappable-seq for XrrA (A), XrrB (B), and XrrC (C). Details are the same as those described in the Fig. 3 legend. Small RNA genes are represented by green arrows. (D) Predicted folding of XrrC. (E) Predicted folding of XrrD. (F) Sequence alignment of XrrC in B. anthracis Sterne and Ames. Nucleotides detected with sequence diversity are marked by “#.”

When analyzing other AtxA-binding sites, we realized that three sites were detected with an upregulated TSS but lacked protein-coding genes nearby (Fig. 4A to C). Two of these TSSs were at the position of small RNAs previously annotated as sRNA1 and sRNA2 (16) and recently annotated as XrrB and XrrA (25). To reveal the length of the transcripts starting from these TSSs, we conducted 3′ rapid amplification of cDNA ends (RACE) (40) and determined their transcription termination sites (TTSs) (Table 3). The TTSs of XrrA and XrrB were within a few nucleotide differences from those in a previous report (25). In addition to the two small RNAs, we also identified an additional 95-bp small RNA from the TSS at position 141844 of pXO1 in the Ames ancestor strain (GenBank accession no. NC_007322.2). We named this small RNA XrrC, according to the nomenclature for other small RNAs encoded by pXO1. As with XrrA, XrrC was also present in the 35-kb pathogenicity island on pXO1.

**TABLE 3:** Annotation of small RNA on pXO1

Small RNA	Start position	End position	Strand	Size (bases)
XrrA	131385	131565	+	181
XrrB	105924	105705	−	220
XrrC	141844	141938	+	95
XrrD	105408	105494	+	87

Approximately 500 bp downstream of the TSS of XrrB, we also identified another TSS with a high level of transcription and heading toward the TSS of XrrB. This TSS was transcribed at the same level in both the WT and Δ*atxA* strains; thus, this was not regulated by AtxA. However, considering the possibility of an overlap of the transcribed region with that of XrrB, we also used 3′ RACE to determine the TTS of this TSS. This region transcribed an 87-bp small RNA, which was short enough to avoid an overlap of the transcription of XrrB. We named this small RNA XrrD. Although XrrD was not regulated by AtxA, the constitutive high level of transcription suggests that this has some functions.

Compared with *xrrA* and *xrrB*, *xrrC* and *xrrD* were short but had long poly(T) regions at the end of their transcripts. The predicted folding of XrrC and XrrD showed a simple structure consisting of a few stem-loops (Fig. 4D and E). In particular, XrrC was predicted to have a symmetric structure, suggesting structure-specific interactions with other RNA-binding proteins such as Hfq homologs (41). Both XrrC and XrrD are highly conserved in the pXO1 plasmid of B. anthracis strains, but we found that 4 bases were changed from GAAA to the reverse complement TTTC at the third stem-loop structure from the TSS of XrrC in the Ames-derived strains. This change may have been caused by an inversion between the flanking inverted repeats that were suggested to form the stem-loop structure (Fig. 4F). The sequence of the loop may not affect the possible function of XrrC.

We constructed single knockouts of the eight direct regulons of AtxA and conducted reverse transcription-quantitative PCR (RT-qPCR) of the three toxin genes to confirm whether these direct regulons affected the transcription of the three toxin genes under the high-CO_2_/bicarbonate conditions (15% CO_2_ and 0.8% NaHCO_3_) (Table 4; Fig. S3). We observed a drastic decrease in the transcript levels of all three toxin genes in the Δ*atxA* strain, as expected. Several of the regulon knockout strains showed an increase or decrease in the transcription levels of *pagA*, *lef*, and *cya*, but none of them were sufficient to explain the decrease observed with the Δ*atxA* knockout, except for those of the *pagA* and *lef* knockouts, which lost the signal for the deleted toxin genes, as expected.

**TABLE 4:** B. anthracis strains

Strain	Description	Reference
34F2	B. anthracis vaccine strain (pXO1^+^ pXO2^−^)	67
BYF10078	34F2 with 1× FLAG tag at the C terminus of *atxA*	This work
BYF10054	34F2 Δ*atxA*	This work
BYF10081	34F2 Δ*xrrA*	This work
BYF10080	34F2 Δ*xrrB*	This work
BYF10107	34F2 Δ*xrrC*	This work
BYF10082	34F2 Δ*xrrA* Δ*xrrB*	This work
BYF10109	34F2 Δ*xrrA* Δ*xrrC*	This work
BYF10108	34F2 Δ*xrrB* Δ*xrrC*	This work
BYF10110	34F2 Δ*xrrA* Δ*xrrB* Δ*xrrC*	This work
BYF10008	34F2 Δ*pagA*	This work
BYF10009	34F2 Δ*lef*	This work
BYF10121	34F2 ΔGBAA_RS28950	This work
BYF10122	34F2 ΔGBAA_RS29020	This work
BYF10123	34F2 ΔGBAA_RS29065	This work

10.1128/mSystems.00291-21.3 FIG S3 RT-qPCR of three toxin genes for knockout strains of AtxA direct regulons. Download FIG S3, PDF file, 0.9 MB. Copyright © 2021 Furuta et al. 2021 Furuta et al. https://creativecommons.org/licenses/by/4.0/ This content is distributed under the terms of the Creative Commons Attribution 4.0 International license.

In total, we detected five protein-coding genes and three small RNAs as the direct regulons of AtxA. We also concluded that *cya*, one of the three toxin genes of B. anthracis, was not directly regulated by AtxA under these culture conditions. Several binding sites were not correlated with the upregulation of nearby TSSs, but such transcription factor binding without an apparent function has been frequently observed in other species (42, 43).

### Characterization of small RNAs directly regulated by AtxA.

**FIG 5:**
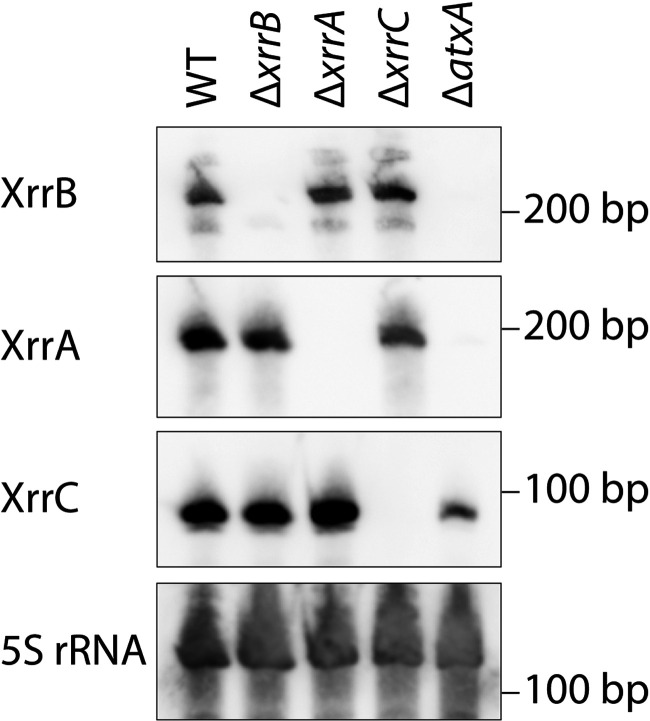
Northern blotting of small RNAs. Probes specific for each small RNA and 5S rRNA were hybridized to whole RNA extracted from each strain grown under the high-CO_2_/bicarbonate conditions (15% CO_2_ and 0.8% NaHCO_3_).

For the three small RNAs directly regulated by AtxA, RNA isolated from WT, Δ*atxA*, and individual small RNA-null mutant strains were analyzed by Northern blotting (Fig. 5). Single major bands of each small RNA were present with the size expected from TSSs and TTSs detected using 3′ RACE (Table 3), indicating that no significant posttranscriptional digestion had occurred. XrrC showed a small amount of expression even in the Δ*atxA* strain, although expression of XrrA and XrrB was not detected. XrrB had two additional minor bands. The results of 3′ RACE of XrrB showed a weaker TTS at position 105739 of pXO1 in the Ames ancestor strain (GenBank accession no. NC_007322.2), which theoretically produces a 186-bp transcript and explains the band corresponding to the smaller transcript of XrrB; no other TTSs could explain the band corresponding to the larger transcript.

**FIG 6:**
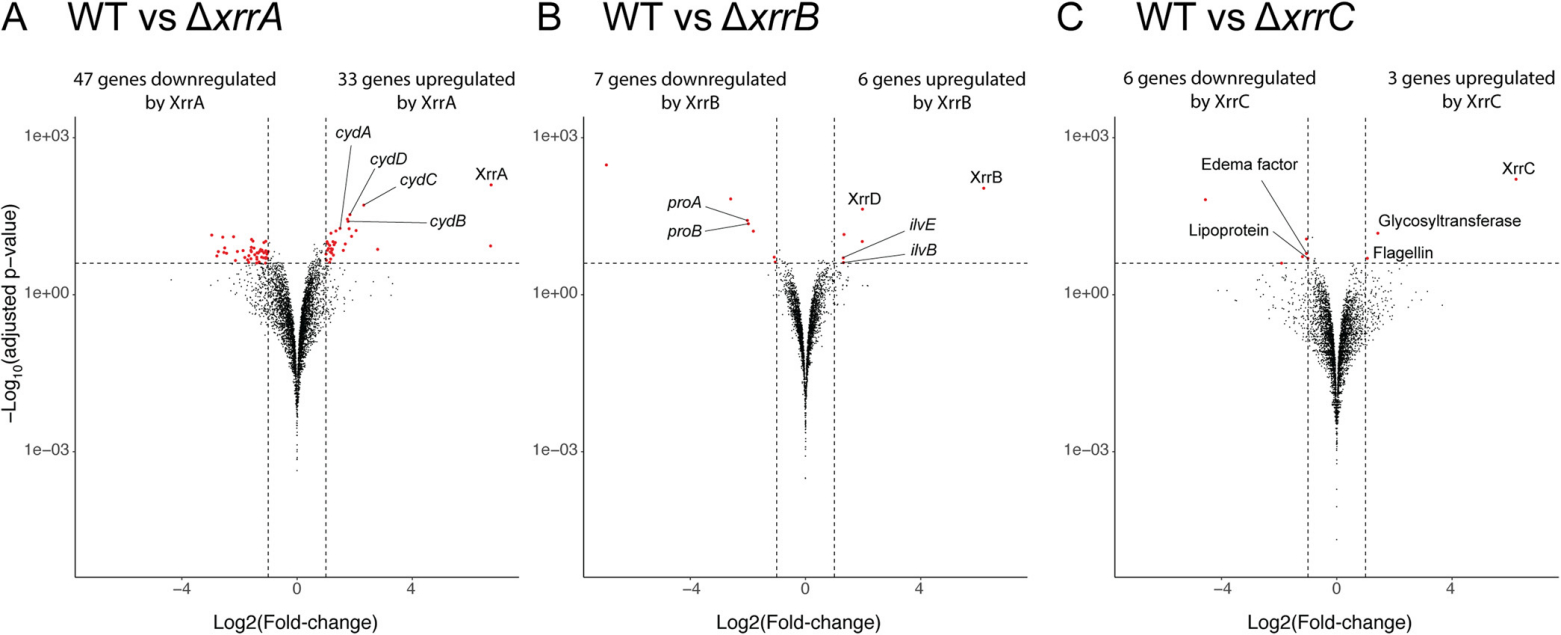
Volcano plots of transcriptome results. (A) Comparison between the WT and Δ*xrrA* strains. (B) Comparison between the WT and Δ*xrrB* strains. (C) Comparison between the WT and Δ*xrrC* strains.

To determine whether these small RNAs regulate other genes in *trans*, RNA-seq was conducted for each single-knockout strain (Fig. 6). Most of the differentially expressed genes detected were located on the chromosome. As in a previous report (25), we observed the same tendency that XrrA regulates a larger number of genes than XrrB. In the Δ*xrrC* strain, nine genes had a significant change in transcription, which was closer to the number found in the Δ*xrrB* strain. There were few overlaps in the differentially expressed genes detected in the small RNA knockout strains, suggesting that they are independent of each other. As we also conducted RNA-seq for the Δ*atxA* strain, we compared the lists of genes with significant transcriptional changes and analyzed the overlap to calculate how much of the AtxA-regulated gene expression was explained by the effect of the small RNAs. The proportions of genes that overlapped those detected in the Δ*atxA* strain were 58/80 (73%), 8/13 (62%), and 8/9 (89%) for the Δ*xrrA*, Δ*xrrB*, and Δ*xrrC* strains, respectively. This explained 71/718 (10%) of the total genes detected in the Δ*atxA* strain (Data Set S2).

10.1128/mSystems.00291-21.7 DATA SET S2 Differentially expressed genes in transcriptome analyses of each strain. Download Data Set S2, XLSX file, 0.08 MB. Copyright © 2021 Furuta et al. 2021 Furuta et al. https://creativecommons.org/licenses/by/4.0/ This content is distributed under the terms of the Creative Commons Attribution 4.0 International license.

We analyzed gene ontology (GO) term enrichment and found that aerobic electron transport chain, heme biosynthetic process, and oxidoreductase activity-related genes were enriched in the Δ*xrrA* strain, and genes related to valine and isoleucine biosynthetic processes were highly represented in the Δ*xrrB* strain (Table 5). XrrA was found to upregulate the genes in the *cyd* operon by 2- to 5-fold, which encodes proteins for the terminal oxidase cytochrome *bd*-I (Data Set S2). RNApredator analysis (44) of the binding target of XrrA identified *cydB* with a high rank of sequence similarity among B. anthracis mRNAs; thus, XrrA might regulate the aerobic respiratory chain by direct interaction. Two genes of small *c*-type cytochromes were previously reported to regulate the expression of toxin genes (45), but none of them were significantly regulated by XrrA. XrrB was found to upregulate the expressions of *ilvEB* and *proAB* by 2-fold and 4-fold, respectively; these genes encode proteins involved in the metabolism of branched-chain amino acids and arginine and proline, respectively (Data Set S2). Therefore, transcriptional regulation by XrrB may affect the amino acid balance in B. anthracis under high-CO_2_/bicarbonate conditions (15% CO_2_ and 0.8% NaHCO_3_). No gene ontology term enrichment was detected for the Δ*xrrC* strain, but genes with annotations such as flagellin, glycosyltransferase, and lipoprotein, all of which have membrane-related functions, were affected by XrrC (Data Set S2). XrrC was also found to downregulate the expression of *cya* (edema factor) but by only 2-fold.

**TABLE 5:** Gene ontology term enrichment of genes upregulated by XrrA/XrrB

sRNA and GO term	Fold enrichment	FDR
XrrA		
Biological process		
Aerobic electron transport chain	69.87	4.04E−03
Cellular respiration	19.96	9.38E−03
Energy derivation by oxidation of organic compounds	18.23	1.12E−02
Generation of precursor metabolites and energy	11.52	1.11E−02
Respiratory electron transport chain	41.92	2.62E−03
Electron transport chain	19.96	1.02E−02
Heme biosynthetic process	46.58	5.45E−03
Pigment biosynthetic process	34.93	2.14E−03
Pigment metabolic process	33.54	1.92E−03
Heme metabolic process	44.13	3.29E−03
Porphyrin-containing compound metabolic process	39.92	1.87E−03
Tetrapyrrole metabolic process	34.93	1.87E−03
Porphyrin-containing compound biosynthetic process	41.92	1.97E−03
Tetrapyrrole biosynthetic process	38.11	1.83E−03
Molecular function		
Oxidoreductase activity, acting on diphenols and related substances as donors, oxygen as an acceptor	78.6	2.50E−02
Oxidoreductase activity, acting on diphenols and related substances as donors	78.6	1.25E−02

XrrB		
Biological process		
Valine biosynthetic process	>100	3.91E−02
Valine metabolic process	>100	2.87E−02
Isoleucine biosynthetic process	>100	3.47E−02
Branched-chain amino acid biosynthetic process	>100	3.29E−02
Branched-chain amino acid metabolic process	>100	3.65E−02
Isoleucine metabolic process	>100	2.60E−02

To further analyze whether small RNAs affect the induction of toxin genes, double- and triple-knockout strains of three small RNAs were constructed, and the transcription levels of toxin genes were measured by RT-qPCR. As reported previously (25), double knockout of XrrA and XrrB showed an approximately 4-fold decrease in *lef* expression. A number of other combinations of small RNAs produced changes in the transcription of toxin genes, but these did not explain the difference in transcription found between the WT and Δ*atxA* strains (Fig. S3).

## DISCUSSION

In this paper, we conducted genome-wide analyses of both AtxA-binding sites and TSSs in the B. anthracis 34F2 strain (pXO1^+^ pXO2^−^). Only eight AtxA-binding sites were identified with statistically significant changes in the transcription levels of TSSs, although more than 300 TSSs were associated with significant changes in their transcription levels in the presence of AtxA. Comparison of these results led to the identification of five genes and three small RNAs that were highly probable to be direct regulons of AtxA. Among these, a new small RNA, named XrrC, was discovered in the pathogenicity island on plasmid pXO1. Transcriptome analysis found that these small RNAs regulated genes related to the aerobic electron transport chain, the heme biosynthetic process, and amino acid metabolism, suggesting some functions in controlling the physiology of the cell during growth under high-CO_2_/carbonate conditions (15% CO_2_ and 0.8% NaHCO_3_).

Genome-wide *in vivo* detection revealed only 11 AtxA-binding sites, which was much less than expected based on the number of AtxA-regulated genes observed in previous reports and our transcriptome analysis (16–19). This suggests that most of the AtxA regulons are indirectly regulated by AtxA. Notably, most of the binding sites were found within the region of the pathogenicity island on pXO1, which is currently defined as the region flanked by IS*1627* (positions 116085 to 163674 of pXO1 in the Ames ancestor strain [GenBank accession no. NC_007322.2]). Recent reports that described small RNAs of B. anthracis have suggested extending the region (positions 104771 to 163674) to include additional small RNAs (16, 25). The current definition includes 7/11 of the AtxA-binding sites, while the suggested definition includes 10/11 sites. We therefore suggest that these binding sites are enriched in the pathogenicity island of pXO1. This is a reasonable observation considering the acquisition of the whole genomic island by horizontal gene transfer. Targets of AtxA would be restricted to local genes in the pathogenicity island of pXO1 for the activation of the pathogenicity of B. anthracis, and transcriptional changes in genes in other regions of the chromosome and the plasmids would be regulated by AtxA indirectly.

The detection of AtxA-binding sites enabled comparison of AtxA binding *in vivo* between the high-CO_2_/bicarbonate conditions (15% CO_2_ and 0.8% NaHCO_3_) and low-CO_2_/bicarbonate conditions (no additional CO_2_ or bicarbonate), where AtxA lacked binding under the low-CO_2_/bicarbonate conditions. The absence of bicarbonate was previously shown not to affect the binding of AtxA to the sequence of the *pagA* promoter *in vitro* (24); thus, the difference in AtxA binding may be explained by changes in AtxA itself. One of the candidates for such changes is the phosphorylation state of AtxA, which is known to affect AtxA activity, but it is not yet clear whether the phosphorylation of both histidine residues of AtxA depends on the concentration of CO_2_/bicarbonate. The fraction of AtxA with phosphorylated H199 and dephosphorylated H379 may increase under high-CO_2_/bicarbonate conditions, and AtxA may then begin to bind to the target regions. Measurement of the fractions of nonphosphorylated, monophosphorylated, and diphosphorylated AtxA under high- and low-CO_2_/bicarbonate conditions would reveal the mechanism of AtxA binding specific to high-CO_2_/bicarbonate conditions in more detail.

Although transcription factors with a small number of regulons tend to have higher binding specificities in Escherichia coli and Bacillus subtilis (46), we could not identify AtxA-binding sites with apparent DNA-binding motifs. DNA bending was also calculated and showed higher distributions than the other regions without AtxA binding (see Fig. S1 in the supplemental material), but several other genomic regions showed the same or even a higher level of bending than that of AtxA-binding sites; thus, this cannot be the only factor determining the binding specificity of AtxA. As discussed previously (24), AtxA binding is highly likely to target multiple factors, such as the DNA sequence, DNA bending, stem-loop formation, and DNA topology. The previously suggested DNA-binding model of AtxA showed the binding of the two DNA strands at both ends of the AtxA dimer; thus, specific short motifs or distantly positioned DNA structures may be recognized by AtxA for binding (22). Another possibility would be an interaction of AtxA with products of its direct regulons, including both proteins and small RNAs, to bind DNA target sites with various specificities. With the AtxA-binding sites revealed in this work, analyses such as screening molecules that interact with AtxA and solving a cocrystal structure of AtxA together with a target DNA region would reveal more detail of the binding mechanism of AtxA (47, 48).

Notably, one of the toxin genes, *cya*, was not detected as a direct regulon, although the other two, *pagA* and *lef*, were clearly detected with both AtxA binding and TSS elevation, as expected. We speculated that these genes were regulated by one of the direct regulons of AtxA; however, no single-knockout strain showed a significant decrease of *cya* as in the Δ*atxA* strain, suggesting a more complex gene regulatory network such as complementation of *cya* regulation by more than one direct or indirect regulon, considering the fact that most promoters are regulated by more than one factor, at least in E. coli (49). It is also possible that genes with weak AtxA binding and/or weak upregulation of transcription may regulate *cya* or that *cya* itself may be regulated by such weak AtxA binding.

We newly detected the 95-bp-long XrrC, in addition to XrrA and XrrB, as a direct regulon of AtxA. Transcriptomes of knockout strains of these small RNAs showed that genes in several categories were enriched by XrrA and XrrB, but no such enrichment was observed with XrrC (Fig. 6). As discussed previously (25), these small RNAs might also regulate gene expression at the translational level. Many small RNAs are known to bind to mRNA and regulate translation, rather than transcription, from the target mRNA (50). The binding of small RNAs to the target sequence was often imperfect and as small as 6 to 7 nucleotides; thus, it is difficult to speculate on the interaction between target genes and small RNAs using only transcriptomic and informatic analyses. Many small RNAs bind to the Hfq protein in Gram-negative species, but it is not yet clear whether Hfq homologs are required for small RNA activity in Gram-positive species (51–53). These small RNAs may interact with one or some of the three Hfq proteins of B. anthracis (54–56) or other proteins to gain some functions or for secretion (57). Thus, for further analysis of these small RNAs in B. anthracis, it will be necessary to analyze the interacting molecules by methods such as RNA pulldown assays (58).

AtxA is also known to regulate genes on the pXO2 plasmid, which we did not analyze in this work because of the use of the 34F2 strain (pXO1^+^ pXO2^−^). AtxA is known to indirectly regulate the *cap* operon, which is required for capsule formation, via positive regulation of *acpA* and *acpB* (11). A fully virulent strain lost its capsule-forming ability when either *atxA* alone or both *acpA* and *acpB* were deleted. It was also shown that AtxA itself induces the expression of the *cap* operon without AcpA and AcpB, although the induction level is lower than that by AcpA and AcpB (19, 59). Therefore, it would be important to analyze the binding of AtxA to the promoters of *acpA*, *acpB*, and the *cap* operon to fully understand the mechanism of capsule formation of B. anthracis. AtxA was also shown to form a heteromultimer with AcpA (19), which may alter the specificity and binding affinity of AtxA or may sequester AtxA. Thus, it would also be important to study the behavior of AtxA for its regulons in the presence of pXO2. Another example of genes on pXO2 upregulated by AtxA is *skiA*. The upregulation of *skiA* by AtxA results in the downregulation of sporulation activity (60). The amino acid sequence of SkiA has similarity to that of the signal sensor domain of BA2291, one of the sporulation sensor histidine kinases of B. anthracis (61). It is suggested that SkiA induced by AtxA under toxin-inducing conditions titrates away the phosphate signals from BA2291, resulting in the downregulation of sporulation (60, 61). One thing to note is that the direct AtxA regulons detected in our work included GBAA_pXO1_0148, formerly annotated as pXO1-118, which was also reported to have similarity to BA2291 and to regulate sporulation activity (61). Although the nucleotide sequences of the promoter regions of *skiA* and GBAA_pXO1_0148 had no similarity, it would be important to analyze whether *skiA* induction was also mediated by direct binding of AtxA.

We noted differences in the results of transcriptome analyses compared to previous research (19, 25). For the Δ*xrrA* and Δ*xrrB* strains, we detected more genes with significant changes in their transcription levels (80 and 13, respectively) than in a previous report that conducted an analysis of knockout strains of *xrrA* and *xrrB* (50 and 1, respectively), with little agreement for gene contents (25). For AtxA regulons detected in the transcriptome analysis of Ames Δ*atxA* Δ*acpA* Δ*acpB* and the strain complemented with AtxA, regulons on pXO1 matched well with our results, while those on the chromosome showed statistically insignificant changes in our results. We used the same genetic background (pXO1^+^ pXO2^−^), growth medium (nutrient broth yeast extract [NBY] medium), and CO_2_ concentration (15%) as the ones used in the previous report of the B. anthracis transcriptome (16), whereas the other studies used a fully pathogenic strain (pXO1^+^ pXO2^+^), a different growth medium (Casamino Acids broth), and 5% CO_2_. Because of these differences, the transcriptomic profiles among our study and the other studies cannot be directly compared. The most widely used *in vitro* growth conditions for B. anthracis include 5% CO_2_, which is close to the normal host environment. This allows an evaluation of infection under a host-like environment. Since the host environment changes with the progression of infection, it would be of interest to investigate gene expression under such conditions. For instance, inhalational anthrax can cause dyspnea (62, 63), which could result in hypoxemia and hypercapnia (64, 65). Whereas we performed transcriptomic profiling under a high-CO_2_ environment, analysis under other host-like environments during infection or directly from the infected host (66) would provide insightful information.

In conclusion, we revealed the regulons under the direct control of AtxA by using two different approaches: detection of the direct binding of AtxA by ChIP-seq and detection of the upregulation of TSSs by Cappable-seq. These regulons consisted of five protein-coding genes, including *pagA* and *lef*, and three small RNA-coding genes, *xrrA*, *xrrB*, and the newly discovered *xrrC*. These results revealed the first layer of the gene regulatory network for the pathogenicity of B. anthracis. More studies of the target specificity of AtxA, the mechanism of transcription of *cya*, the target and function of small RNAs, and the presence/absence of AtxA direct regulons on pXO2 are required for a complete understanding of this complex gene regulatory network.

## MATERIALS AND METHODS

### Strains and plasmids.

B. anthracis 34F2 (pXO1^+^ pXO2^−^) (67) derivative strains were constructed as previously described (68, 69). Strains are listed in Table 4. Strain BYF10078 was constructed by inserting a 1× FLAG tag as a translational fusion at the C terminus of the *atxA* gene on pXO1 to minimize differences in the copy numbers of the AtxA protein.

For the growth conditions, we used the same ones as those in a previous study of the transcriptome of the pXO1^+^ pXO2^−^ strain (16). Bacteria were cultured in NBY medium (0.8% [wt/vol] nutrient broth, 0.3% [wt/vol] yeast extract, and 0.5% [wt/vol] glucose) supplemented with 0.8% NaHCO_3_ with shaking in a CO_2_ incubator with 15% CO_2_ at 37°C as the high-CO_2_/bicarbonate conditions, whereas NBY medium without supplementation with NaHCO_3_ and shaking in a usual dry shaker at 37°C was used as the low-CO_2_/bicarbonate conditions.

### ChIP-seq and ChIP-qPCR.

Sample preparation for ChIP-seq was conducted with three biological replicates as described previously, with some modifications (70, 71). Single colonies of B. anthracis 34F2 (pXO1^+^ pXO2^−^) and the derivative strain BYF10078, which expresses AtxA-1× FLAG from pXO1, on brain heart infusion (BHI) plates were inoculated in 2 ml medium for culture under the high-CO_2_/bicarbonate conditions (15% CO_2_ and 0.8% NaHCO_3_) at 37°C for 3 h. The culture was then diluted 100-fold into 30 ml medium for culture under the high-CO_2_/bicarbonate conditions (15% CO_2_ and 0.8% NaHCO_3_) at 37°C for 3 h. Cultures were pelleted by centrifugation at 8,000 × *g* for 10 min at room temperature, followed by resuspension with 3 ml NBY medium with 0.8% NaHCO_3_. Formaldehyde (220 μl of 16% [vol/vol]; Thermo Fisher Scientific, Waltham, MA) was added, the mixture was incubated for cross-linking at room temperature for 30 min with rotation using a HulaMixer (Thermo Fisher Scientific, Waltham, MA), 360 μl of 2.5 M glycine was added, and the mixture was incubated for quenching at room temperature for 15 min with rotation as described above. Samples were pelleted by centrifugation at 12,000 rpm for 3 min at room temperature, followed by resuspension in 1 ml of 1× phosphate-buffered saline and centrifugation under the same conditions.

Pellets were resuspended with 200 μl lysis buffer (20 mM HEPES-KOH [pH 8.0], 50 mM KCl, 10% glycerol, 0.1% NP-40) supplemented with cOmplete Mini protease inhibitor cocktail tablets (Roche, Basel, Switzerland) and sonicated using a Covaris S2 instrument (Covaris, Woburn, MA) with a 20% duty cycle, an intensity of 5, 200 cycles per burst, and 3 cycles of 60 s. Sonicated samples were centrifuged at 12,000 rpm for 20 min at 4°C after the addition of 750 μl lysis buffer. After the addition of 10 μl of 1 M Tris-HCl (pH 8.0) and 20 μl of 5 M NaCl, 50 μl of the sample was removed as an unenriched control. The remaining sample volume was then combined with 40 μl of anti-FLAG M2 affinity gel (Merck, Darmstadt, Germany), equilibrated with the lysis buffer, and incubated with rotation at 4°C overnight.

The solution was centrifuged at 5,000 × *g* for 1 min at room temperature, and the pellets were resuspended with 1 ml of wash buffer 1 (10 mM Tris-HCl [pH 8.0], 500 mM NaCl, 1 mM EDTA, 0.1% NP-40), followed by centrifugation for 1 min at room temperature and resuspension with 1 ml of wash buffer 2 (10 mM Tris-HCl [pH 8.0], 150 mM NaCl, 1 mM EDTA, 0.1% NP-40). Samples were centrifuged at 5,000 × *g* for 1 min at room temperature and then resuspended with 1 ml of Tris-EDTA (TE) buffer (50 mM Tris-Cl [pH 8.0], 10 mM EDTA). Samples were again centrifuged at 5,000 × *g* for 1 min at room temperature, resuspended with 100 μl of elution buffer (50 mM Tris-Cl [pH 8.0], 10 mM EDTA, 1% SDS), and then reacted with 0.5 μl of 10 mg/ml of proteinase K at 65°C overnight. For the unenriched sample, 50 μl of 2× elution buffer (100 mM Tris-Cl [pH 8.0], 20 mM EDTA, 2% SDS) was added and reacted with proteinase K as described above. After centrifugation at 5,000 × *g* for 1 min at room temperature, the supernatant was purified by using a Minelute PCR purification kit (Qiagen, Hilden, Germany) and eluted with 50 μl buffer EB from the kit.

Sequence libraries were prepared using the NEBNext Ultra II DNA library prep kit for Illumina (New England BioLabs, Ipswich, MA) and sequenced using the MiSeq platform (Illumina, San Diego, CA) for 101-bp, single-end reads.

Sequence reads were applied for adapter trimming and quality filtering using Trim Galore! version 0.6.4_dev with the option “-a GATCGGAAGAGCACACGT,” followed by mapping to the genome sequence of the B. anthracis Ames ancestor strain (RefSeq assembly accession no. GCF_000008445.1, with manual modification for sequence differences in *xrrC*) using BWA-mem 0.7.17 (72). The mapping data were analyzed using the R package csaw 1.22.1 (73, 74) with a 158-bp fragment length and a 50-bp window width. Windows with >5.5 average counts per million reads were used for further analysis, with a false discovery rate (FDR) of ≤0.05 as a statistical threshold.

Detected enriched regions were confirmed by ChIP-qPCR of the sample used for ChIP-seq using PowerUp SYBR green master mix (Thermo Fisher Scientific, Waltham, MA) in at least triplicate. The primers used are listed in Table S2 in the supplemental material.

Motif analysis of the AtxA-binding regions was performed using MEME 5.0.5 (31) with the following parameters: “-dna -oc anr -mod anr -nmotifs 3 -minw 4 -maxw 20 -objfun classic -revcomp.” The intrinsic curvature of the regions was calculated using the stand-alone version of bend.it 1.0 (32) with default parameters.

### Cappable-seq.

Cappable-seq was conducted as described previously (33). B. anthracis 34F2 (pXO1^+^ pXO2^−^) and Δ*atxA* cells were grown and pelleted as described above for ChIP-seq, with three biological replicates, followed by resuspension with 100 μl of boiling lysis buffer (2% SDS, 16 mM EDTA [pH 8.0], 20 mM NaCl), with incubation at 100°C for 5 min. RNA was extracted from the heated resuspensions using TRIzol (Thermo Fisher Scientific, Waltham, MA). After the removal of DNA using a DNA-free DNA removal kit (Thermo Fisher Scientific, Waltham, MA), RNA samples were capped with 3′ desthiobiotin-GTP (New England BioLabs, Ipswich, MA) specifically at the 5′ end with triphosphate using vaccinia capping enzyme (New England BioLabs, Ipswich, MA) and fragmented with 10× T4 polynucleotide kinase buffer (New England BioLabs, Ipswich, MA), with 3′ phosphates removed using T4 polynucleotide kinase (New England BioLabs, Ipswich, MA). Capped RNA was enriched using hydrophobic streptavidin magnetic beads (New England BioLabs, Ipswich, MA) twice and then decapped using RppH (New England BioLabs, Ipswich, MA). For all procedures for RNA purification with AMPure RNAClean XP (Beckman Coulter, Brea, CA), a 1.8× volume of beads was used together with a 1.5× volume of ethanol to avoid the loss of small RNAs.

Using enriched and unenriched samples, sequencing libraries were prepared using the NEBNext multiplex small RNA library prep set for Illumina (New England BioLabs, Ipswich, MA) using a half-concentration of adapters. Sequencing was conducted using MiSeq (Illumina, San Diego, CA) with a 101-bp read length.

For the analysis of sequence reads, the adapter sequence was removed using cutadapt 2.1 with the option “-a AGATCGGAAGAGCACACGTCTGAACTCCAGTCAC,” followed by mapping to the genome sequence of the B. anthracis Ames ancestor (RefSeq assembly accession no. GCF_000008445.1, with manual modification for sequence differences in *xrrC*) using Bowtie2 2.3.5 (75) with the option “-L 16.” Coverage of the first base of mapped reads was calculated for each position by considering strands and was then compared between enriched and unenriched samples using EdgeR 3.30.3 (76, 77) with the threshold of an FDR of ≤0.05 and a log fold change of ≥1 to remove positions not derived from TSSs such as those of processed rRNA. Strain-specific TSSs were detected by the same calculation between the WT TSS and the Δ*atxA* TSS with a threshold FDR of ≤0.05. Multiple TSSs on the same strand within a 5-bp distance were clustered into a single position with the highest average coverage per million reads. Sequences around TSSs were analyzed with WebLogo 3 (78) to visualize the conserved pattern in the promoter region.

### Annotation, folding prediction, and target prediction of small RNAs.

For 3′ RACE, RNA isolated from biological duplicates of cultures of the WT and Δ*atxA* strains under the high-CO_2_/bicarbonate conditions (15% CO_2_ and 0.8% NaHCO_3_) was directly applied for the synthesis of cDNA ligated with the adapters at both ends using the NEBNext multiplex small RNA library prep set for Illumina (New England BioLabs, Ipswich, MA) without fragmentation. For PCR amplification of the cDNA, primers specific to the 5′ end of each small RNA (Table S2) were used instead of the SP primer to amplify cDNA derived from small RNAs. PCR was conducted for 17 cycles, and the purified libraries were sequenced with MiSeq (Illumina, San Diego, CA) for 50-bp, paired-end reads. Reads were mapped to the B. anthracis Ames ancestor (RefSeq assembly accession no. GCF_000008445.1, with manual modification for sequence differences in *xrrC*) using BWA-backtrack 0.7.17 (79). Positions of the 5′ ends of each read were collected and used for the assignment of TTSs.

mFold (80) was used for the prediction of the folding of small RNAs. RNApredator 1.55 (44) was used for the prediction of the target of small RNAs.

### Northern blotting for small RNA.

Northern blotting for small RNA was conducted as previously described (81). The complement strands of each small RNA and 5S rRNA were cloned into pSPT18 and used for probe preparation using the digoxigenin (DIG) RNA labeling kit (Roche, Basel, Switzerland) with T7 RNA polymerase. Isolated RNAs (5 μg) from the WT and single-knockout strains of each small RNA grown under the high-CO_2_/bicarbonate conditions (15% CO_2_ and 0.8% NaHCO_3_) were mixed with the same volume of loading buffer (80% Hi-Di formamide, 5 mM EDTA [pH 8.0], 10 μg/ml xylene cyanol, 10 μg/ml bromophenol blue), heated at 95°C for 5 min, and then immediately placed on ice. Samples were loaded onto a 15% urea-polyacrylamide gel with Dynamarker RNA Low II easy load (BioDynamics Laboratory Inc., Tokyo, Japan) and run in 1× Tris-borate-EDTA (TBE) buffer at 200 V and 20 mA for 1 h. RNA was blotted onto a Hybond-N^+^ nylon membrane (Cytiva, Marlborough, MA) using a Trans-Blot SD semidry transfer cell (Bio-Rad, Hercules, CA) at 20 V and 200 mA for 1 h. Membrane-bound RNA was cross-linked with 1,200 mJ of UV using the UVLink 1000 cross-linker (Analytik Jena, Jena, Germany). The membrane was preheated with ULTRAhyb buffer (Thermo Fisher Scientific, Waltham, MA), and specific probes were added. Probes were heated at 50°C for XrrC detection, whereas probes for other small RNAs and 5S rRNA were used at 68°C. The membrane was washed, and blocking solution (Roche, Basel, Switzerland) was added. Signals were detected using CDP-Star (Tropix, Bedford, MA).

### RNA-seq.

WT and knockout strains were grown under the high-CO_2_/bicarbonate conditions (15% CO_2_ and 0.8% NaHCO_3_), and RNA was extracted as described above. rRNA was removed by the do-it-yourself (DIY) method with a set of biotin-modified primers that were redesigned to include rRNAs of B. anthracis as a target (82) (Table S2). After fragmentation with a divalent cation and heat and removal of 3′ phosphates using T4 polynucleotide kinase (New England BioLabs, Ipswich, MA), RNA was used for library preparation with the NEBNext multiplex small RNA library prep set for Illumina (New England BioLabs, Ipswich, MA). The library was sequenced with HiSeq X (Illumina, San Diego, CA) for 151-bp, paired-end reads by Macrogen Japan Corp. (Tokyo, Japan).

Reads were trimmed using Trim Galore! 0.6.4_dev with the option “-a AGATCGGAAGAGA” and mapped to the B. anthracis Ames ancestor (RefSeq assembly accession no. GCF_000008445.1, with manual modification for sequence differences in *xrrC*) using BWA-mem 0.7.17 (72), and tags were counted with htseq-count (83). Counts were analyzed with DESeq2 1.28.1 (84). Genes with an adjusted *P* value of <0.0001 and an absolute fold change value of ≥2 were considered differentially expressed genes. Gene ontology term enrichment was examined for the functional analysis of differentially expressed genes using OmicsBox version 1.4 (BioBam Bioinformatics, Valencia, Spain) and the GO enrichment analysis tool (85).

RT-qPCR of toxin genes was conducted with a Power SYBR green RNA-to-*C_T_* 1-step kit (Thermo Fisher Scientific, Waltham, MA). The primers used are listed in Table S2; *gatB-yqeY* was used for normalization (86).

### Data availability.

All sequence data produced by Illumina sequencers and processed data were deposited in the Gene Expression Omnibus under accession no. GSE167871.
