# Supplementary material for: Direct Regulons of AtxA, the Master Virulence Regulator of Bacillus anthracis
Source: mSystems. 2021 Jul 20;6(4):e00291-21. doi: 10.1128/mSystems.00291-21 (PMC8407390; doi:10.1128/mSystems.00291-21)
Supplement: FIG S1 [file msystems.00291-21-sf001.pdf]

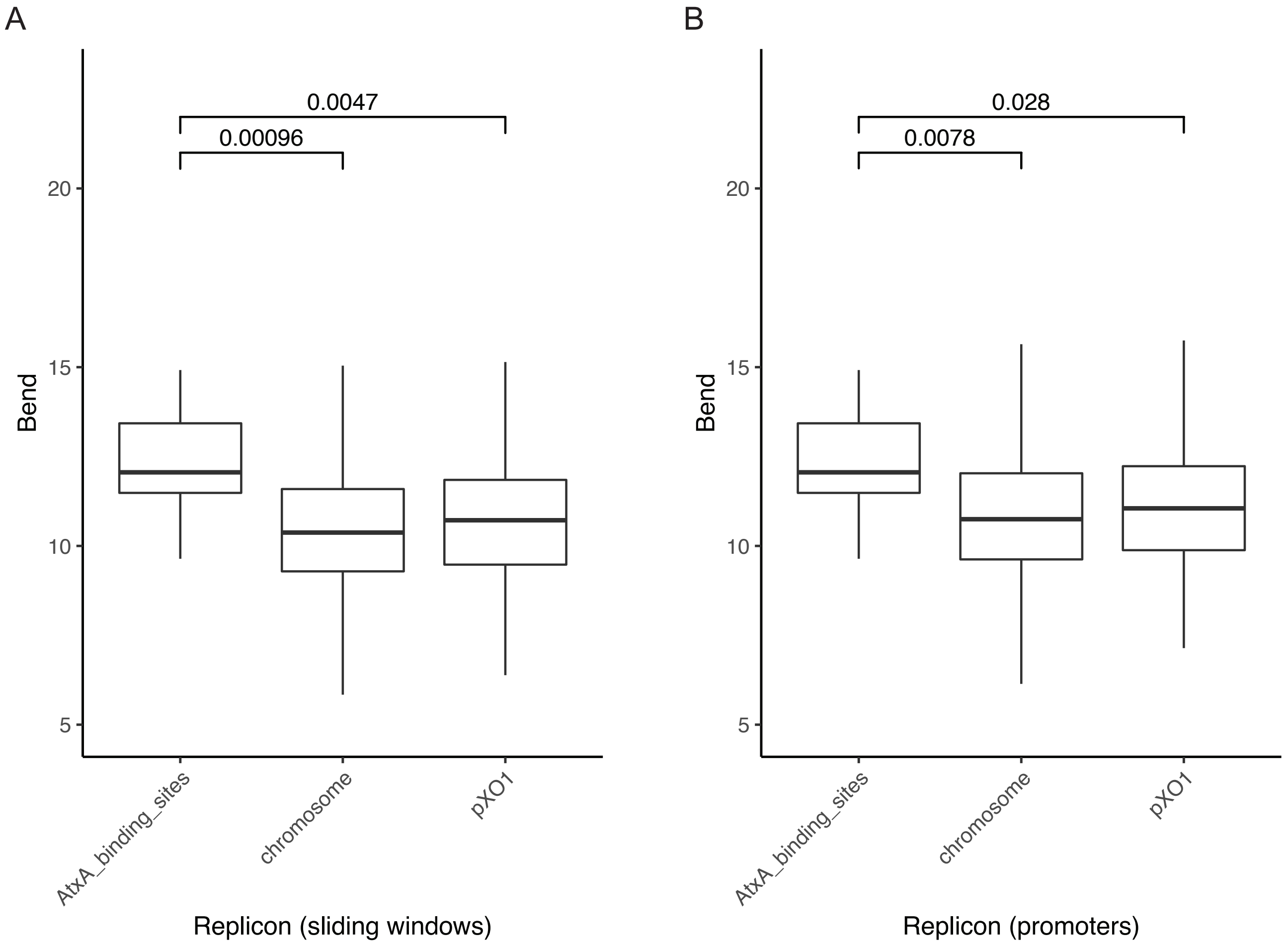

Fig S1. Comparison of DNA bending. (A) Comparison between AtxA binding region and 500-bp sliding window of each amplicon. (B) Comparison between AtxA binding region and upstream region of TSS.
