# Supplementary material for: Direct Regulons of AtxA, the Master Virulence Regulator of Bacillus anthracis
Source: mSystems. 2021 Jul 20;6(4):e00291-21. doi: 10.1128/mSystems.00291-21 (PMC8407390; doi:10.1128/mSystems.00291-21)
Supplement: TABLE S1 [file msystems.00291-21-st001.pdf]

**Table S1. Validation of TSS with previous reports.**

| Replicon | Gene name | Promoter | Experimental TSS | This work WT | This work Δ*atxA* | Reference | Comments |
|---|---|---|---|---|---|---|---|
| pXO1 | *pagA* | P1 | 143721(+) | 143721 (+) | N/D | (2) | |
| | | P2 | 143754 (+) | 143747 (+) | N/D | (2) | |
| | *lef* | P1 | 151815 (-) | 151815 (-) | N/D | (3) | |
| | | P2 | N/D | 151805 (-) | N/D | This work. | |
| | *cya* | P1 | 122547 (+) | 122548 (+) | 122548 (+) | (3) | |
| | | P2 | N/D | 122583 (+) | 122583 (+) | This work. | |
| | *atxA* | P1 | 129291 (-) | 129291 (-) | 129291 (-) | (3) | |
| | | P2 | 129934 (-) | 129934 (-) | 129934 (-) | (34) | |
| | | P3 | 129876 (-) | N/D[a] | N/D | (34) | |
| | *gerXB* | P1 | 137122 (+) | N/D | N/D | (35) | Expressed during germination process |
| | *xrrA* | | 131385 (+) | 131385 (+) | N/D | (25) | |
| | *xrrB* | | 105925 (-) | 105924 (-) | N/D | (25) | |
| Chromosome | *abrB* | | 41187 (-) | 41187 (-) | 41187 (-) | (36) | |
| | *sap* | | 896422 (+) | N/D | 896421 (+) | (37) | |
| | *eag* | | 899545 (+) | N/D | 899535 (+) | (37) | Expressed during stationary phase |
| | *alr* | P1 | 239696 (+) | N/D | N/D | (38) | Expressed during sporulation process |
| | | P2 | 239623 (+) | N/D | N/D | (38) | Expressed during sporulation process |
| | *alo* | | 3088142 (-) | 3088148 (-) | 3088148 (-) | (39) | |

a. N/D, Not detected.
