# Supplementary material for: Direct Regulons of AtxA, the Master Virulence Regulator of Bacillus anthracis
Source: mSystems. 2021 Jul 20;6(4):e00291-21. doi: 10.1128/mSystems.00291-21 (PMC8407390; doi:10.1128/mSystems.00291-21)
Supplement: TABLE S2 [file msystems.00291-21-st002.pdf]

Table S2. Primers.

Name	Sequence	Comment
PrYF727	AAAGCTACAAACTCTGAAATTTGTAAATTG	For ChIP-qPCR of PL3 region.
PrYF728	CAACGATGATTGGAGATAGAGTATTCTTT	For ChIP-qPCR of PL3 region.
PrYF1105	TCAATATGATGTAGAGCGGAGTTTG	For ChIP-qPCR of AtxA_BS_01 region.
PrYF1106	AGTGTTGTTTGTTTTTATCGATAGGG	For ChIP-qPCR of AtxA_BS_01 region.
PrYF1107	GAAACGTAATTTTACAAAGTGTCAAAGG	For ChIP-qPCR of AtxA_BS_02 region.
PrYF1108	AGCATTTTTGTTAGGGGAAGAATAATAG	For ChIP-qPCR of AtxA_BS_02 region.
PrYF1109	ACTGATAATTCGAATCACGTGACG	For ChIP-qPCR of AtxA_BS_03 region.
PrYF1110	CACTGTCATACTGCGGAATAACTTC	For ChIP-qPCR of AtxA_BS_03 region.
PrYF1111	GTGTATTTAGAATAATTAAGGGGTTATAGCTG	For ChIP-qPCR of AtxA_BS_04 region.
PrYF1112	GTGTCTTTATTTGGGGTTTTAATTTCCAG	For ChIP-qPCR of AtxA_BS_04 region.
PrYF1113	AATCTAGCTTAGGATTAATGAATGTTACTG	For ChIP-qPCR of AtxA_BS_05 region.
PrYF1114	TTTGTTGCTTCCATAATTCCACTCC	For ChIP-qPCR of AtxA_BS_05 region.
PrYF1115	GCGGATTTAGAAAATTGTTGCATCAC	For ChIP-qPCR of AtxA_BS_06 region.
PrYF1116	CTCTTGAACTATAACATTCAACAACATAGC	For ChIP-qPCR of AtxA_BS_06 region.
PrYF1117	TCACCTTTTTATGTAAAAATATTCCTTAACG	For ChIP-qPCR of AtxA_BS_07 region.
PrYF1118	CCACTGAATATTAAATTTTAATAGGGGTCAC	For ChIP-qPCR of AtxA_BS_07 region.
PrYF1119	GTTTTATGCCATAATAGGTTTATAGTTTTTAAGC	For ChIP-qPCR of AtxA_BS_08 region.
PrYF1120	GTGTGTTCTAGACATGATGAGTTTTCC	For ChIP-qPCR of AtxA_BS_08 region.
PrYF1121	ATTGCGAAAATTATAAGCGTTTCTTG	For ChIP-qPCR of AtxA_BS_09 region.
PrYF1122	ATCATGAACACCGGCTCCATG	For ChIP-qPCR of AtxA_BS_09 region.
PrYF1123	GCCCATTTATCATTTGCATTCCAC	For ChIP-qPCR of AtxA_BS_10 region.
PrYF1124	CATTCTGGATAGTCAATAAATAGATTACGG	For ChIP-qPCR of AtxA_BS_10 region.
PrYF1125	CCTTGCATGAATTACCTTTAAGGAG	For ChIP-qPCR of AtxA_BS_11 region.
PrYF1126	TGAGAGCATGCTCATATTGTGTTAC	For ChIP-qPCR of AtxA_BS_11 region.
PrYF1259	ACCAAAGGAGGTTTAAGAATGACTAG	For ChIP-qPCR of upstream of *cya* .
PrYF1260	GCATTTACTTCTATAGCCTGTGAGG	For ChIP-qPCR of upstream of *cya* .
PrYF946	AATGATACGGCGACCACCGAGATCTACACGTTCAGAGTTCTACAGTCCGACGATCATGTTGTTGAATGTTATAGTTCAAG	For 3'-RACE of *xrrA* .
PrYF945	AATGATACGGCGACCACCGAGATCTACACGTTCAGAGTTCTACAGTCCGACGATCGGATACTTTGTTTACCTCTGTTAAG	For 3'-RACE of *xrrB* .
PrYF1104	AATGATACGGCGACCACCGAGATCTACACGTTCAGAGTTCTACAGTCCGACGATCATAGTTTTTAAGCAATTATGATTGAAAACTATAAG	For 3'-RACE of *xrrC* .
PrYF947	AATGATACGGCGACCACCGAGATCTACACGTTCAGAGTTCTACAGTCCGACGATCAATAGTTTTTAAACTTAAAAAGGTACAAG	For 3'-RACE of *xrrD* .
PrSM21	AGCTGGTCGTGAAGACCTTG	For RT-qPCR of *gatB-Yqey* .
PrSM22	CGGCATAACAGCAGTCATCA	For RT-qPCR of *gatB-Yqey* .
PrYF756	AGTGCATGCGTCGTTCTTTG	For RT-qPCR of *pagA* .
PrYF757	ACCCATTGTTTCAGCCCAAG	For RT-qPCR of *pagA* .
PrYF754	TATCTTGCCAGCATCCGTTG	For RT-qPCR of *lef* .
PrYF755	ACAGCTTTATGCACCGGAAG	For RT-qPCR of *lef* .
PrYF729	AGGTAGATTTATAGAAAAAAACATTACGGG	For RT-qPCR of *cya* .
PrYF730	GCTGACGTAGGGATGGTATT	For RT-qPCR of *cya* .
PrYF976	TTCACCTTTCCCTCACGGTACTGGTTCGCTATCGGTCAC	For 23S rRNA depletion. 5′ biotinylated.
PrYF977	AAGTCGCTGGCTCATTATACAAAAGGCACGCCGTCACC	For 23S rRNA depletion. 5′ biotinylated.
PrYF978	TCGGGGAGAACCAGCTATCTCCGGGTTTGATTGGC	For 23S rRNA depletion. 5′ biotinylated.
PrYF979	TGGTGGCTGCTTCTAAGCCAACATCCTGGT	For 23S rRNA depletion. 5′ biotinylated.
PrYF980	GTACAGGAATATTAACCTGATTTCCATCGACTACGCCTGTCGGCCTCG	For 23S rRNA depletion. 5′ biotinylated.
PrYF981	CCTACCTGTGTCGGTTTGGGGTACGG	For 23S rRNA depletion. 5′ biotinylated.
PrYF982	CTTACCCGACAAGGAATTTCGCTACCTTAGGACGG	For 23S rRNA depletion. 5′ biotinylated.
PrYF983	TGACGAGCCGACATCGAGGTGCCAAAC	For 23S rRNA depletion. 5′ biotinylated.
PrYF984	TAGGGACCGAACTGTCTCACGACGTTCTAAACCCA	For 23S rRNA depletion. 5′ biotinylated.
PrYF985	ACGTCCTTCATCGCCTTTTACTGCCAAGGCATCCGCC	For 23S rRNA depletion. 5′ biotinylated.
PrYF986	GCTTACACACCCGGCCTATCAACGTGGTGGTCT	For 23S rRNA depletion. 5′ biotinylated.
PrYF987	GCCAAGGCATCCACCGTGCGCCCTTA	For 23S rRNA depletion. 5′ biotinylated.
PrYF988	ACAACTGGTACACCAGAGGTACGTCCATCCCGG	For 23S rRNA depletion. 5′ biotinylated.
PrYF989	TCGACTTGCATGTGTTAAGCATGCCGCTAGCGT	For 16S rRNA depletion. 5′ biotinylated.
PrYF990	GAAGATTCCCCACTGCTGCCTCCCGTAG	For 16S rRNA depletion. 5′ biotinylated.
PrYF991	ATTACCGCGGCTGCTGGCACGGAGTT	For 16S rRNA depletion. 5′ biotinylated.
PrYF992	TGGACTACCAGGGTATCTAATCCTGTTCGATCCCC	For 16S rRNA depletion. 5′ biotinylated.
PrYF993	ATGCTCCGCCGCTTGTGCGGGCCCCCGTCAATT	For 16S rRNA depletion. 5′ biotinylated.
PrYF994	TCACGACACGAGCTGACGACAGCCATG	For 16S rRNA depletion. 5′ biotinylated.
PrYF995	AGTGTGTACAAGGCCCGGGAACGTATTCACC	For 16S rRNA depletion. 5′ biotinylated.
PrYF996	AGGAGGTGATCCAGCCGCAGCTTCCGCTACGGCTACCTTG	For 16S rRNA depletion. 5′ biotinylated.
PrYF997	TTCCGTGTTCGGCATGGGAACGGGTGTGGCCTC	For 5S rRNA depletion. 5′ biotinylated.
PrYF998	TCACAGGGAGAAGCTCCCGACTACCATCGGCG	For 5S rRNA depletion. 5′ biotinylated.
PrYF999	CTGAGTTCGGGAAGGGGTCAGGTGGGTCCAA	For 5S rRNA depletion. 5′ biotinylated.
PrYF1000	GAGACCCCACACTACCATCGGCGATACGTCGT	For 5S rRNA depletion. 5′ biotinylated.
