# Supplementary material for: Direct Regulons of AtxA, the Master Virulence Regulator of Bacillus anthracis
Source: mSystems. 2021 Jul 20;6(4):e00291-21. doi: 10.1128/mSystems.00291-21 (PMC8407390; doi:10.1128/mSystems.00291-21)
Supplement: FIG S2 [file msystems.00291-21-sf002.pdf]

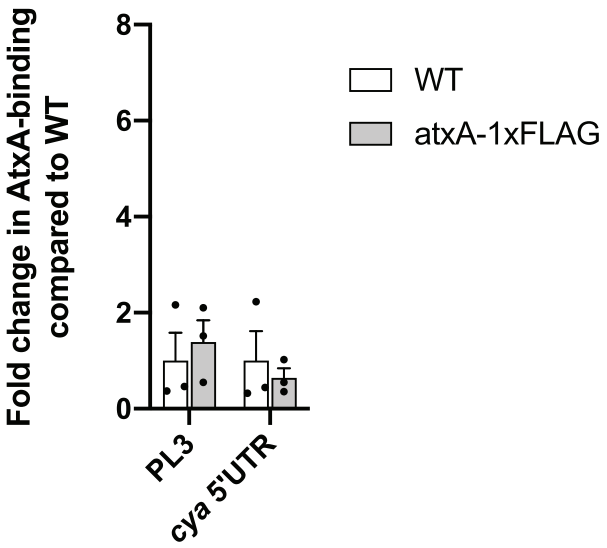

Fig S2. ChIP-qPCR of the upstream of *cya* for ChIP samples isolated from cells grown under high CO_2/bicarbonate levels.
