# Supplementary figures and images for: Direct Regulons of AtxA, the Master Virulence Regulator of Bacillus anthracis
Source: mSystems. 2021 Jul 20;6(4):e00291-21. doi: 10.1128/mSystems.00291-21 (PMC8407390; doi:10.1128/mSystems.00291-21)

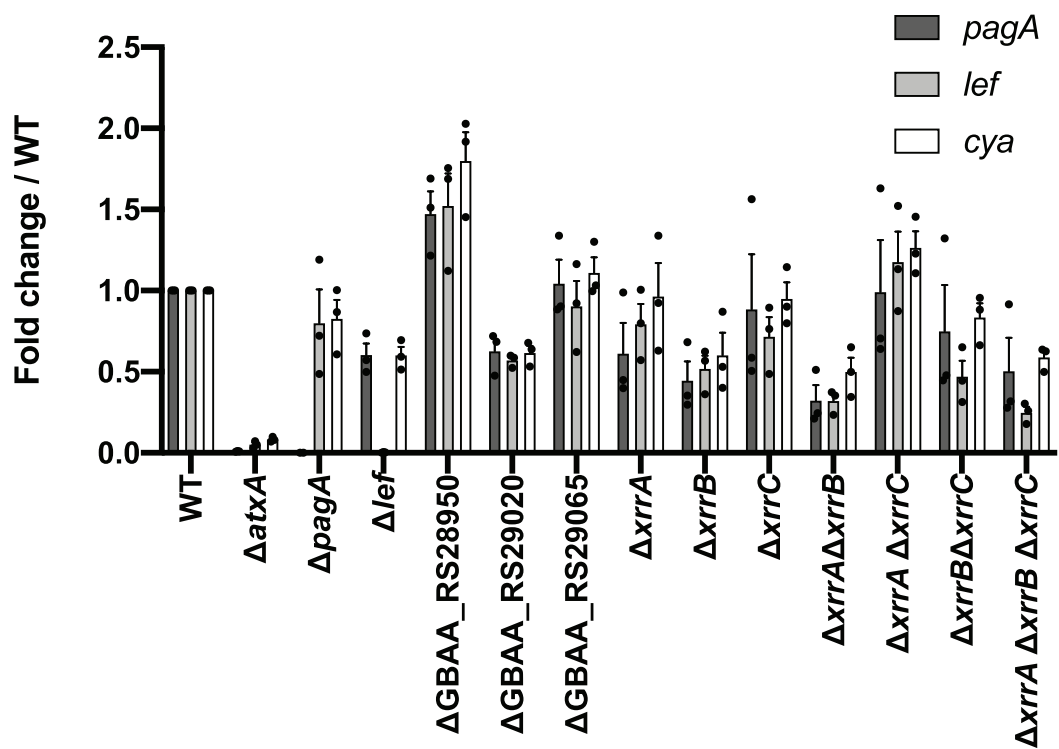

Fig S3. RT-qPCR of three toxin genes for knockout strains of AtxA direct regulons.

Supplement: FIG S3 [file msystems.00291-21-sf003.pdf]
